# Ankle Joint Dorsiflexion Reference Values in Non-Injured Youth Federated Basketball Players: A Cross-Sectional Study

**DOI:** 10.3390/ijerph191811740

**Published:** 2022-09-17

**Authors:** Cristina Adillón, Montse Gallegos, Silvia Treviño, Isabel Salvat

**Affiliations:** 1 Faculty of Medicine and Health Sciences, Department of Medicine and Surgery, Institut d’Investigació Sanitària Pere Virgili, Universitat Rovira i Virgili, 43003 Tarragona, Spain; 2 Health Department, Catalan Basketball Federation, 08018 Barcelona, Spain

**Keywords:** adolescent, pediatrics, ankle, range of motion, basketball

## Abstract

(1) Background: The aim of the present study was to establish ankle joint dorsiflexion reference values among youth federated basketball players. (2) Methods: Cross-sectional study. The participants were basketball players who belonged to youth basketball developmental teams (female and male) from under-12 (U12) to under-17 (U17) categories. Ankle joint dorsiflexion range of motion was evaluated with the weight-bearing lunge test through the Leg Motion system. The distance achieved was recorded in centimeters. (3) Results: 693 basketball players who met the eligibility criteria and volunteered to participate were included in the study. The mean (SD) of ankle joint dorsiflexion was 10.68 (2.44) cm and the reference values were: excessive hypomobility < 6.09 (0.54) cm; hypomobility 6.09 (0.88) cm–8.43 (0.77) cm; normal 8.44 (0.77)–13.11 (0.79) cm; hypermobility 13.11 (0.74)–15.44 (0.86) cm; and excessive hypermobility >15.44 (0.86) cm. (4) Conclusions: This study provides ankle joint dorsiflexion reference values in youth basketball players from 12 to under 17 years old.

## 1. Introduction

The ankle joint is a complex of articulations whose main actions are to allow dorsiflexion (flexion) and plantar flexion (extension) of the foot. Injuries in these areas are very frequent and can result in limited mobility due to the involvement of the ankle and foot during locomotion [1].

A reduction in ankle joint dorsiflexion has been identified as a risk factor for sustaining several common lower-extremity injuries during physical activity [2,3], including lateral ankle sprain [3,4], plantar fasciopathy [5,6], medial tibial stress syndrome and knee anterior cruciate ligament injury [3,7,8,9], iliotibial band syndrome [3,8], patellofemoral pain syndrome [3,7,8], and patellar tendinopathy [3,7,10]. Moreover, it is often identified as a point of emphasis during lower-extremity rehabilitation [3,11] especially in conditions related to ankle sprains [3] either as an injury risk factor or as a common sequela after injury [12,13]. Inadequate restoration of ankle dorsiflexion may limit functional activities [3] and increase the risk of developing recurrent ankle sprain [3,14]. Furthermore, mobility development and maintenance are recommended as a part of any training program in order to ensure that players are able to perform the ranges of motion required for their discipline [15].

A reduction in ankle joint dorsiflexion is also associated with kinematic alterations of both the hip and knee [8], including the dynamic knee valgus [16], which is related to several knee disorders. Decreased ankle dorsiflexion may reduce the amount of available force that can be absorbed when landing through this joint and, consequently, an increase in the force that may be absorbed by the rest of the joints (the knee, hip, and trunk) [17]. Alterations in hip and knee movements, in the frontal and transverse plane, caused by the lack of mobility in the ankle may contribute to the development of anterior knee pain or other kinetic chain imbalances that could lead to overuse injuries [18]. 

Despite the fact that there is no universal consensus on the value at which ankle joint dorsiflexion can be considered limited [19], Baumbach et al. [20] have recently suggested that dorsiflexion less than 30 degrees should be regarded as restricted. Based on Baumach et al.’s study, Searle et al. [21] have established and categorized the normative ankle dorsiflexion ranges for young adults into excessive hypermobility, hypermobility, normal, hypomobility, and excessive hypomobility. Hypermobility is a term that describes cases in which a joint is able to move beyond its normal range of motion (ROM) [22], whereas hypomobility describes cases in which a joint is unable to achieve its normal ROM, usually secondary to passive ligamentous/capsular restrictions or muscle stiffness of the tibialis anterior, extensor digitorum longus, or peroneus tertius [1]. 

In the clinical setting, it is important to assess ankle joint dorsiflexion range of motion [8,21]. Currently, there are several methods to measure ankle dorsiflexion described in the literature [19]: the distance from the toe to wall [23], the degrees of tibial inclination [24], or the conventional goniometry [24]. The last one has been reported to be highly unreliable and to have poor reproducibility [24] because it has been assessed in the open-chain position [12,25]. Therefore, techniques that involve weight bearing are increasing in relevance because they are more representative of lower-extremity function during activity [23,26]. However, there is currently no consensus on measuring ankle joint range of motion [27]. The most commonly used reference values for joint ROM are measured by a universal goniometer using non-weight-bearing tests [19,28,29,30].

Clinically, the weight-bearing dorsiflexion test should be used frequently by healthcare providers who are responsible for preventing and treating lower-extremity injuries to assess ankle dorsiflexion range of motion as it provides consistent and repeatable results among one or more clinicians [31,32]. Unfortunately, there is insufficient evidence describing either normal ankle joint mobility in centimeters or cut-off values to determine the normal joint amplitude values according to age range [8,29]. The determination of reference values by age range is important because, according to Souci et al. [29], the mean values of range of motion of all joints decrease with age in both men and women. 

For all the above-mentioned reasons, the aim of this study was to establish baseline ankle joint dorsiflexion values in federated basketball players aged 12 to less than 17 years since normal joint amplitude values are necessary to assess joint motion and evaluate the degree of deterioration after injury, and whether preventive measures are necessary to avoid injury. 

## 2. Materials and Methods

### 2.1. Study Design

This cross-sectional study was conducted from October 2018 to February 2020. The study adhered to the tenets of the Declaration of Helsinki and received ethical approval from the local institutional review board (Pere Virgili Institute; Ref. CEICm: 123/2018). The study protocol was registered with ClinicalTrials.gov ID: NCT04796753.

Informed consent was obtained from the children and their parents or guardians to participate in the study.

### 2.2. Participants

The participants were basketball players who belonged to youth basketball developmental teams in the under-12 (U12), under-14 (U14), under-16 (U16), and under-17 (U17) categories. All participants were recruited by means of simple random sampling from the Catalan Basketball Federation during the 2018–2019 season and the 2019–2020 season. The participants were classified according to gender and age as stipulated by the rules determined annually for the respective official competitions. The study was carried out in the facilities of each club. The inclusion criteria were being aged ≥10 and ≤17 at testing and actively competing during the study. Subjects were excluded if they had sustained any type of injury in the lower limbs before screening; presented any injury (overuse or acute) at the time of testing; if they had any oncological, psychological, and/or psychiatric illnesses; or if they did not attend on the day of the assessment. The final sample size was a convenience sample, determined by the number of players who agreed to participate voluntarily.

### 2.3. Outcomes

**Figure 1:**
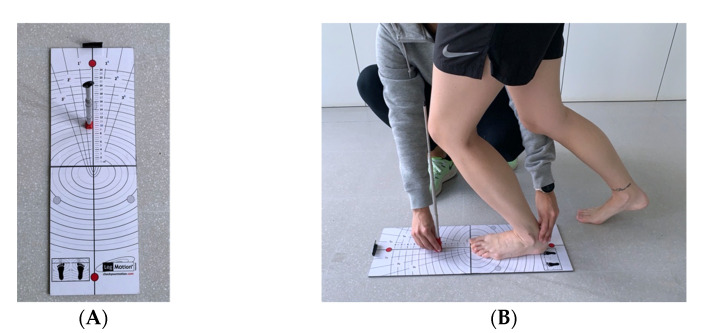
(**A**) The LegMotion system, (**B**) Weight-bearing lunge test, conducted using the LegMotion system.

Primary outcome measures: Ankle joint dorsiflexion was evaluated with the weight-bearing lunge test through the Leg Motion system (LegMotion, your Motion^®^, Albacete, Spain) (Figure 1A) [31,32]. This test has been validated for this purpose. All players were familiarized beforehand with the test procedure. It was performed on the same day and at the same time of day (6.00 to 8.00 p.m.).

Secondary outcome measures: Age, age categories, gender, weight, height, wingspan, and body mass index were recorded. The presence of hypermobility was evaluated by Beighton’s criteria (scores of ≥7 points out of a total of 9 points were considered hypermobile) [33], and lower-limb dominance was observed with the criteria described by Harris on foot dominance [34].

### 2.4. Data Sources and Procedure 

Ankle joint dorsiflexion was evaluated with the weight-bearing lunge test through the Leg Motion system (Figure 1B). Subjects were instructed to try to bring the knee to touch the metal rod (initially placed at a distance of 10 cm) without lifting the heel off the ground. The distance achieved was recorded in centimeters [31,32].

All participants allowed three practice trials for the weight-bearing lunge test. They were placed in a standing position with one foot on the Leg Motion^®^ platform with the second toe over the central line. The other foot was required to be placed outside the platform resting on the floor. In this position, the subjects were instructed to try to bring the knee to touch the metal rod (initially placed at a distance of 10 cm) without lifting the heel off the ground system [31]. If the subject could maintain heel and knee contact with the metal rod, the evaluator moved the metal rod away from the knee. 

The maximum range of motion was defined as the maximum distance (in centimeters) from the second toe to the metal rod without losing contact between the rod and the knee for three seconds, and without lifting the heel off the ground. All measurements were completed with the participant barefoot, performing all tests first with one limb and then with the other.

Three attempts were performed for each side and the highest value was selected for data analysis. The attempt was discarded if the subject lifted the heel off the ground or did not follow the standards for performing the test.

### 2.5. Bias

To minimize observation bias, the researcher in charge of analyzing the results did not know the hypothesis of the study and used measuring instruments with previously established evaluation criteria.

### 2.6. Statistical Analysis

Statistical analysis was performed using SPSS (Statistical Package for the Social Sciences for Windows, version 26.0). The normality of each variable was confirmed by means of the Shapiro–Wilk test. Normally distributed data for continuous variables were summarized with means and standard deviations (SD). Qualitative variables were described as absolute frequencies and percentages. A multifactor ANOVA will be performed to analyze whether gender or age category influences ankle joint dorsiflexion. Levene’s test will be performed first in order to apply it.

The reference interval (95% confidence interval) and the reference limits will be established using the reference range calculations defined by Searle et al. [21]:

- Excessive Hypomobility: <−2 · SD;
- Hypomobility: −2 · SD < x < −1 · SD;
- Normal: −1 · SD < x < 1 · SD;
- Hypermobility: 1 · SD < x < 2 · SD;
- Excessive Hypermobility: >−2 · SD.

For all tests, *p*-values were two-sided. A value 0 < 0.05 was considered significant.

## 3. Results

### 3.1. Description of Sample

**Figure 2:**
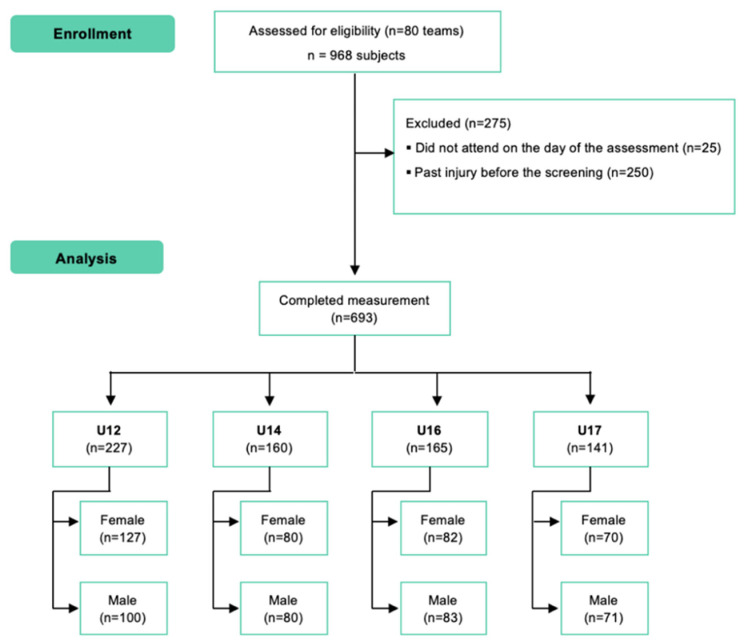
Flow diagram.

Nine hundred and sixty-eight players were recruited to participate in the study. Two hundred and fifty were excluded from the study because of past injury prior to screening and twenty-five did not attend on the day of the assessment (Figure 2). In the end, six hundred and nighty-three youth basketball players who met the eligibility criteria and volunteered to participate were included in the study. The mean (SD) age is 13.36 (2.17) and 52% of the participants are female. 

Descriptive characteristics for anthropometric data are reported in Table 1. All data were found to be normally distributed. As would be expected based on maturation, U17 players were taller, heavier, and had a larger wingspan compared to the other players. In all categories, 10% of the players presented generalized hyperlaxity and the right leg was identified as being dominant for 90.30% (*n* = 626) of participants.

### 3.2. Ankle Joint Dorsiflexion

**Figure 3:**
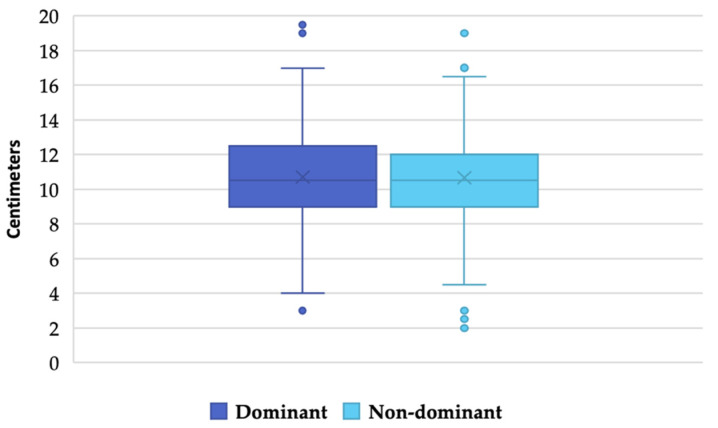
Differences in ankle joint dorsiflexion between dominant leg/nondominant leg.

Although this variable does not follow a normal distribution (0.99; *p* < 0.001; both in the dominant and nondominant leg), as the sample is greater than 100 observations, its distribution can be considered as normal [35]. The mean (SD) for ankle joint dorsiflexion is 10.68 (2.44) cm, with no statistically significant differences between the dominant leg/nondominant leg (*p* = 0.827) (see Figure 3). 

### 3.3. Ankle Joint Dorsiflexion for between-Group Comparisons and Gender of U12 to U17 Basketball

Levene’s median-based testing order was performed in order to compare whether the groups were homogeneous in terms of gender. It can be observed that the *p*-value of the test (0.432) is higher than the significance value 0.05; therefore, it can be concluded that there is homogeneity between the two groups. Similarly, there is also homogeneity between the groups because it can be seen that the *p*-value of the test (0.524) is higher than the significance value 0.05.

The U16 category presented more mobility than the rest of the categories (see Table 2). More detailed statistics are provided for all age groups in Table 3.

### 3.4. Reference Interval and Reference Limits 

For this analysis, the averages of joint range of motion measures from the dominant side were used as the joint range of motion measurement for each subject, because there were no statistically significant differences between the dominant leg/nondominant leg.

The means (SD) of the reference values are: excessive hypomobility, <6.09 (0.54); hypomobility, 6.09 (0.88)–8.43 (0.77); normal, 8.44 (0.77)–13.11 (0.79); hypermobility, 13.11 (0.74)–15.44 (0.86); and excessive hypermobility, >15.44 (0.86). Table 4 shows the confidence intervals for the classification to establish reference ankle joint dorsiflexion values in people from 12 to under 17 years old.

### 3.5. Classification of the Sample According to Reference Values

According to the degree of ankle joint dorsiflexion, 468 participants (67.51%) had normal values, 104 (15.92%) ankle hypermobility, and 18 (2.60%) excessive hypermobility; at the other extreme, 88 (12.71%) had hypomobility and 15 (2.14%) had excessive hypermobility. Detailed values by age categories and gender can be found in Table 5.

No relationship was found between general hypermobility and ankle hypermobility (chi-square, *p* = 0.280).

## 4. Discussion

This study provides data on reference ankle joint dorsiflexion values measured in weight bearing in healthy young people (aged 10 to 17 years) of both genders. It has a large population of 693 participants, which places it among the studies with the largest sample size. Indeed, Souci et al. carried out a study with 674 subjects (aged 2–69 years) [29] and Hallaçeli’s work had 987 subjects (aged 19–32 years) [30]. Although all these studies were conducted in a healthy population, their exclusion criteria do not mention that the participants had not suffered a previous ankle sprain, which is known to limit ankle dorsiflexion [3,36], as occurred in the present study. 

In fact, the studies by Souci et al. [29] and Hallaçeli [30] presented goniometric values without weight loading, whereas in the present study, the stride test with weight loading was used to measure ankle joint dorsiflexion. As Rabin et al. [27] state, the two measurements do not assess the exact same phenomenon, and they should not be used interchangeably as measures of ankle joint dorsiflexion range of motion. In fact, the correlation found between both values is moderate [7,27,29,37,38] with much higher variability by non-weight-bearing goniometric values. 

The weight-bearing lunge is the most widely used test with more concrete and consistent values than goniometry [12]. Our values coincide with the results described by Gonzalo-Skok et al. [39] in a similar study, especially among U14 basketball players, but these results are not comparable with other studies [20,27,29,30,40,41,42], since most of them measure dorsal ankle flexion in angles (degrees) (Table 6). Moreover, the weight-bearing measuring technique is the more accurate measurement because it replicates the position of the ankle during functional activities, such as squatting, jump landing, or stair climbing [27]. The non-weight-bearing measurement may not stress the ankle to its full excursion and, therefore, it may not be sensitive enough under these circumstances [27]. The preferred measurement technique should be determined by the investigator’s/clinician’s specific purpose [27]. 

In fact, the present study was performed with basketball players and this sport has a very high percentage of ankle sprain [36,39,43,44,45] and knee injuries [46]. To prevent and treat these injuries, clinical guidelines and rehabilitation protocols emphasize achieving more degrees of ankle joint dorsiflexion, especially after an ankle sprain. Therefore, it can be very useful to know the reference degrees of ankle joint dorsiflexion, since it is often not possible to count on finding the same degrees as the other ankle, as it may have been previously injured, given the frequency with which this injury occurs in basketball players [36,39,43,44,45]. 

Previous reports have been inconsistent regarding the symmetry of joint range of motion in young healthy individuals [29]. This was perhaps because it was not assessed whether the right or left side was dominant or nondominant. The results of the present study are consistent with the literature; there were no clinically significant differences between the ankle joint dorsiflexion of the dominant or nondominant side. 

The present study not only presents the mean values of ankle dorsiflexion mobility, but also allows the classification of ankle joint dorsiflexion into excessive hypermobility, hypermobility, normal, hypomobility, and excessive hypomobility, and characterizes the differences between gender and between age categories in noninjured youth federated basketball players. Our results agree with those of other studies in the sense that ankle joint dorsiflexion is lower in men than women in all age categories [29,30]. 

Nevertheless, some limitations have been found in this study. It may be debatable whether the values found can be extrapolated to other types of population or countries, because Asian populations have been shown to have greater ankle dorsiflexion compared to the Western population [30]. On the other hand, most of the studies performed measure dorsiflexion with goniometry, unlike the tool used in the present study.

However, the data obtained in the present study might be useful to clinicians in assessing the impact of diseases such as hemophilia, rheumatoid arthritis, or muscular dystrophy on joint mobility [30]. Likewise, these established values may be useful, also, for the evaluation of disorders that have a symmetrical distribution, in case a healthy, unaffected limb is not available for comparison, or it can also be used to follow the evolution of the disease over time.

The values found in this study establish reference values which are not reference values in the pediatric and juvenile population, despite the fact that this group has been described as the most vulnerable to injury [47]. Future studies should assess the relationship between decreased ankle dorsiflexion (excessive hypomobility and hypomobility) and hip and knee kinematic alterations, specifically the dynamic knee valgus. Thus, the classification of ankle mobility established in the present article could be assessed according to its clinical relevance. Further research should investigate sensitivity to change the measurement in response to several therapeutic interventions. 

## 5. Conclusions

In summary, this study provides ankle joint dorsiflexion reference values in youth federated basketball players from 12 to under 17 years old.

## Figures and Tables

**Table 1:** Anthropometric data for between-group comparisons of U12 to U17 basketball players.

Outcomes	U12 (*n* = 227)	U14 (*n* = 160)	U16 (*n* = 165)	U17 (*n* = 141)
Gender ^a^, female	127 (55.95%)	80 (50%)	82 (49.70%)	70 (49.64%)
Weight ^b^, kg	43.97 (8.14)	53.96 (10.91)	65.69 (11.93)	68.30 (13.57)
Height ^b^, cm	154.69 (7.68)	164.04 (9.45)	176.50 (10.32)	177.63 (11.60)
Wingspan ^b^, cm	152.85 (11.14)	164.62 (13.43)	177.16 (11.88)	178.96 (13.62)
BMI ^b^, kg/m^2^	18.27 (2.44)	19.93 (2.86)	21.04 (3.11)	21.60 (3.43)
Hypermobility ^a^	14 (6.17%)	17 (10.63%)	7 (4.24%)	12 (8.51%)
Right-handed ^a^	199 (87.67%)	146 (91.25%)	153 (92.73%)	128 (90.78%)

Abbreviations: BMI, body mass index; m, meter; kg, kilogram; cm, centimeter. Data are reported as ^a^ *n* (%) or ^b^ as mean (standard deviation) % (percentage).

**Table 2:** Assessment of ankle joint dorsiflexion for between-group comparisons of U12 to U17 basketball players.

Gender	Age Categories	Dominant Leg			Nondominant Leg			*p* Value
		Mean (SD)	SE	Interval	Mean (SD)	SE	Interval	
Female	U12 (*n* = 127)	10.54 (2.42)	2.39	[8.15, 12.93]	10.26 (2.55)	2.39	[8.15, 12.93]	0.186
	U14 (*n* = 80)	10.83 (2.36)	2.37	[8.48, 13.17]	11.04 (2.59)	2.35	[8.48, 13.17]	0.294
	U16 (*n* = 82)	11.77 (2.90)	2.08	[9.69, 13.85]	11.38 (1.84)	2.08	[9.69, 13.85]	0.103
	U17 (*n* = 70)	11.28 (2.21)	2.20	[9.07, 13.48]	11.44 (2.11)	2.20	[9.07, 13.48]	0.327
Male	U12 (*n* = 100)	9.21 (2.16)	2.14	[7.07,11.36]	9.40 (2.04)	2.13	[7.07, 11.36]	0.271
	U14 (*n* = 80)	10.81 (2.48)	2.47	[8.34,13.27]	10.74 (2.41)	2.47	[8.34, 13.27]	0.436
	U16 (*n* = 83)	10.97 (2.87)	2.85	[8.12,13.82]	11.09 (2.79)	2.85	[8.12, 13.82]	0.392
	U17 (*n* = 71)	10.70 (2.12)	2.11	[8.58,12.81]	10.71 (2.24)	2.11	[8.58, 12.81]	0.485

Abbreviations: SD, standard deviation; SE, standard error. Values are centimeters of ankle joint dorsiflexion. *p* values were obtained by independent samples student’s *t*-test.

**Table 3:** Ankle dorsiflexion range of motion in different gender and age groups.

	U12		U14		U16		U17	
Joint ROM	Female	Male	Female	Male	Female	Male	Female	Male
No. of subjects	127	100	80	80	82	83	70	71
Mean	10.54	9.22	10.83	10.81	11.77	10.97	11.28	10.70
SD	2.42	2.16	2.36	2.48	2.09	2.87	2.21	2.12
Minimum	4	4	6	5	5	3	5	6.50
25th percentile	9.50	8	9	9.50	10.50	9.50	9.50	9
50th percentile	10.50	9	10.5	10.50	11.50	11	11.50	10.50
75th percentile	12	10.04	13	12	13.50	13	13	12.50
Maximum	19.50	17	17	16	16	19	16.50	15.50

Abbreviations: ROM, range of motion; SD, standard deviation. Values are centimeters of ankle joint dorsiflexion through the Leg Motion system.

**Table 4:** Reference values (centimeters) for Weight-bearing lunge test for 12- to under-17-years-old basketball players.

Gender	Age Categories	Excessive Hypomobility	Hypomobility	Normal	Hypermobility	Excessive Hypermobility
Female	U12	<5.70	5.70–8.12	8.13–12.95	12.96–15.37	>15.37
	U14	<6.11	6.11–8.47	8.48–13.18	13.19–15.54	>15.54
	U16	<7.59	7.59–9.68	9.69–13.86	13.87–15.95	>15.95
	U17	<6.86	6.86–9.07	9.08–13.49	13.50–15.70	>15.70
Male	U12	<4.89	4.89–7.05	7.06–11.38	11.39–13.54	>13.54
	U14	<5.85	5.85–8.33	8.34–13.29	13.30–15.77	>15.77
	U16	<5.23	5.23–8.10	8.11–13.84	13.85–16.71	>16.71
	U17	<6.46	6.46–8.58	8.59–12.82	12.83–14.93	>14.93

Values are centimeters of ankle joint dorsiflexion through the Leg Motion system.

**Table 5:** Classification of the sample according to reference values for between-group comparisons of U12 to U17 basketball players.

Gender	Age Categories	Mean	95% CI	Excessive Hypomobility	Hypomobility	Normal	Hypermobility	Excessive Hypermobility
Female	U12 (*n* = 127)	10.54	10.11–10.96	5 (3.90%)	13 (10.20%)	88 (69.30%)	19 (15.00%)	2 (1.60%)
	U14 (*n* = 80)	10.82	10.30–11.35	1 (1.30%)	11 (13.80%)	54 (67.50%)	12 (15.00%)	2 (2.50%)
	U16 (*n* = 82)	11.77	11.31–12.23	2 (2.40%)	8 (9.80%)	57 (69.50%)	14 (17.10%)	1 (1.20%)
	U17 (*n* = 70)	11.28	10.75–11.81	1 (1.40%)	13 (18.60%)	41 (58.60%)	14 (20.00%)	1 (1.40%)
Male	U12 (*n* = 100)	9.21	8.78–9.64	2 (2.00%)	13 (13.00%)	72 (72.00%)	9 (9.00%)	4 (4.00%)
	U14 (*n* = 80)	10.81	10.25–11.36	1 (1.30%)	11 (13.80%)	53 (66.30%)	11 (13.80%)	4 (5.00%)
	U16 (*n* = 83)	10.97	10.34–11.60	3 (3.60%)	9 (10.80%)	55 (66.30%)	14 (16.90%)	2 (2.40%)
	U17 (*n* = 71)	10.70	10.20–11.20	0 (0%)	10 (14.10%)	48 (67.60%)	11 (15.50%)	2 (2.80%)

Abbreviations: CI, confidence intervals. Data are reported as *n* (%). Values are centimeters of ankle joint dorsiflexion through the Leg Motion system.

**Table 6:** Comparison of mean and normative values for ankle joint dorsiflexion.

Author	No People	Age	Dorsiflexion Mean (SD)	Excessive Hypomobility (<−2 SD)	Hypomobility (−2 to −1 SD)	Normal (−/+ 1 SD)	Hypermobility (+1 to +2 SD)	Excessive Hypermobility (>+2 SD)
**Non-weight-bearing passive**								
Hallaçeli et al. [30] ^1^	987	19–32 y	22.44 (7.16) ^a^	<8.12	8.12–15.28	15.29–29.60	29.61–36.77	>36.77
Souci et al. [29] ^1^	674	9–19 y	16.80 (5.75) ^a^	<5.30	5.30–11.05	11.06–22.55	22.56–28.30	>28.30
Rabin et al. [27] ^1^	43	20–30 y	49.78 (6.40)	<36.98	36.98–43.38	43.39–56.18	58.19–62.58	>62.58
Baumbach et al. [20] ^1^	60	18–35 y	28.13 (6.29)	<15.55	15.55–21.84	21.85–34.42	34.43–40–71	>40.71
**Weight-bearing active**								
Krause et al. [41] ^1^	39	18–35 y	33.29 (7.07) ^a^	<18.80	18.80–25.90	25.91–40.40	40.41–47.00	>47.00
Baumbach et al. [20] ^1^	64	18–35 y	37.77(5.82) ^b^	<26.13	26.13–31.95	31.96–37.77	37.78–43.59	>43.59
Munteanu et al. [42] ^1^	30	19–42 y	39.0 (4.6)	<29.80	29.80–34.30	34.31–43.60	43.61–48.10	>48.10
Konrad et al. [43] ^1^	38	20–26 y	31.5 (6.6)	<18.20	18.20–24.80	24.81–38.10	38.11–44.60	>44.60
Gonzalo-Skok et al. [40] ^2^	15	14–16 y	10.94 (3.44) ^b^	<4.06	4.06–7.50	7.51–14.38	14.39–17.82	>17.82
Adillón et al. ^2^	693	10–17 y	10.68 (2.44)	<6.09	6.09–8.43	8.44–13.11	13.12–15.44	>15.44

Abbreviations: SD, standard deviation; y, years. Values are reported as ^1^ degrees or ^2^ centimeters of ankle joint dorsiflexion, ^a^ averaged from group data, ^b^ averaged from left and right sides.

## Data Availability

The study did not report any data.
